# CSF Aquaporin-4 levels in controls and in idiopathic normal pressure hydrocephalus before and after shunt surgery

**DOI:** 10.1186/s12987-026-00809-2

**Published:** 2026-04-21

**Authors:** Johan Eriksson De Ryst, Sofia Bergström, Sara Mravinacova, David Bäckström, Sara Qvarlander, Anna Månberg, Anders Eklund, Jan Malm

**Affiliations:** 1 Department of Clinical Sciences, Neurosciences, Umeå University, Umeå, 901 87 Sweden; 2 Department of Diagnostics and Intervention, Biomedical Engineering and Radiation Physics, Umeå University, Umeå, 901 87 Sweden; 3 Division of Affinity Proteomics, Department of Protein Science, KTH Royal Institute of Technology, SciLifeLab, Stockholm, Sweden

**Keywords:** Aquaporin 4, Cerebrospinal fluid, Normal pressure hydrocephalus, idiopathic, Glymphatic system

## Abstract

**Background:**

Aquaporin-4 (AQP4) is crucial for brain fluid regulation and glymphatic system function. Idiopathic normal pressure hydrocephalus (INPH) is characterized by impaired CSF flow and is treated with shunt surgery. This study investigated AQP4 levels in INPH patients to explore its role in pathophysiology and as a potential biomarker for shunt response.

**Methods:**

CSF samples from 233 INPH patients and 29 controls were analysed. AQP4 levels were compared between preoperative patients and controls, before and after shunt surgery (110 patients), and between shunt responders and non-responders (204 patients). A bead-based assay was used to measure AQP4, and outcomes were assessed by postoperative changes in maximum gait velocity.

**Results:**

In unadjusted analyses, preoperative AQP4 levels were lower in INPH patients than in controls; however, this difference did not remain after adjustment for pre-analytical and demographic confounders (*p* = 0.87). Postoperative AQP4 levels were higher (median 1646 AU IQR 1347–1976) than preoperative levels (1166 AU IQR: 976–1345; *p* < 0.001) and the magnitude of increase showed a modest correlation with gait improvement (rₛ = 0.22, *p* = 0.022). Shunt responders had lower preoperative AQP4 levels median 1089 AU, IQR 971–1277) than non-responders (median 1213 AU, IQR 1074–1361; *p* = 0.008). Pre-analytical factors, including storage duration and sample processing, were strong determinants of measured AQP4 levels.

**Conclusions:**

CSF AQP4 levels in INPH did not differ from those in controls and were highly sensitive to pre-analytical sample handling. CSF AQP4 levels increased following shunt surgery. A potential prognostic value of CSF AQP4 is suggested but requires further investigation.

## Introduction

Aquaporins (AQPs) are membrane proteins that form channels enabling the free passage of water along osmotic or hydrostatic gradients [1]. They play a crucial role in maintaining fluid homeostasis in the brain [1], with AQP4 being particularly implicated in facilitating cerebrospinal fluid (CSF) flow through the glymphatic system [2–4]. According to this hypothesis, water from the CSF in the subarachnoid space (SAS) enters the arterial perivascular spaces and subsequently flows into the brain’s interstitial fluid (ISF) through the glia limitans, a barrier formed by astrocytic endfeet. This specific transport of water is facilitated by AQP4 channels on astrocytic endfeet. After water has traversed the parenchyma, AQP4 facilitates its efflux from the interstitial fluid (ISF) into CSF within the venous perivascular space, thereby creating a bulk flow that enables the removal of soluble waste products from the interstitial space, completing the glymphatic flow pathway.

INPH is a neurodegenerative disorder with a reported prevalence of up to 3.7% in individuals over the age of 65 [5]. It is characterized by ventricular enlargement and distinctive symmetrical gait disturbance [6, 7]. A key feature of INPH is impaired CSF flow [8], and the condition is treatable by CSF shunting [9]. Reduced glymphatic flow has recently been proposed as a contributing factor in the pathophysiology of INPH [10]. This hypothesis is supported by studies in which intrathecal administration of gadolinium-based contrast agent, followed by serial magnetic resonance imaging (MRI), demonstrated delayed uptake in the brain parenchyma and impaired clearance from the CSF in INPH patients [11, 12].

There are indications that the loss of AQP4 localization in the perivascular wall in both Alzheimer’s disease (AD) [13] and INPH [14–17] contributes to disrupted glymphatic flow. Several studies analysing cortical biopsies from INPH patients have shown a reduction in the normally polarized perivascular expression of AQP4 on astrocytic endfeet [14–17]. Consequently, AQP4 has been hypothesized to be a potential target for future medical treatment of INPH [18]. As AQP4 is in direct contact with CSF, disturbances in its expression should be reflected in its CSF levels. Therefore, CSF AQP4 could serve as a biomarker, for example, for distinguishing INPH from its mimics and predicting the effectiveness of shunt surgery [14, 17–21]. Two studies have investigated AQP4 levels in CSF in the context of INPH. The first included 10 subjects with INPH and found no difference compared with 9 controls [21]. The second study, involving 81 subjects with INPH, failed to detect any AQP4 [22]. To date, no studies have compared AQP4 levels before and after surgery.

In this study, we measured CSF AQP4 levels in 233 patients with INPH and compared these levels to those of age-matched controls (*n* = 29). Additionally, we evaluated how shunt treatment influences CSF AQP4 levels.

## Methods

In summary, this observational study included CSF samples from 233 shunted INPH patients and 29 control subjects. Preoperative CSF was collected from all INPH patients, and 110 of these also had CSF saved from a follow-up lumbar puncture after shunt surgery. All samples were promptly stored in a biobank and analysed simultaneously. Preoperative AQP4 levels were compared with those of age-matched controls. In patients with longitudinal samples, preoperative levels were also compared with postoperative levels. Furthermore, we compared AQP4 levels between shunt responders and non-responders and assessed correlations between changes in AQP4 levels and changes in maximal gait velocity (V_max_) after surgery.

### Study population and controls

**Fig. 1:**
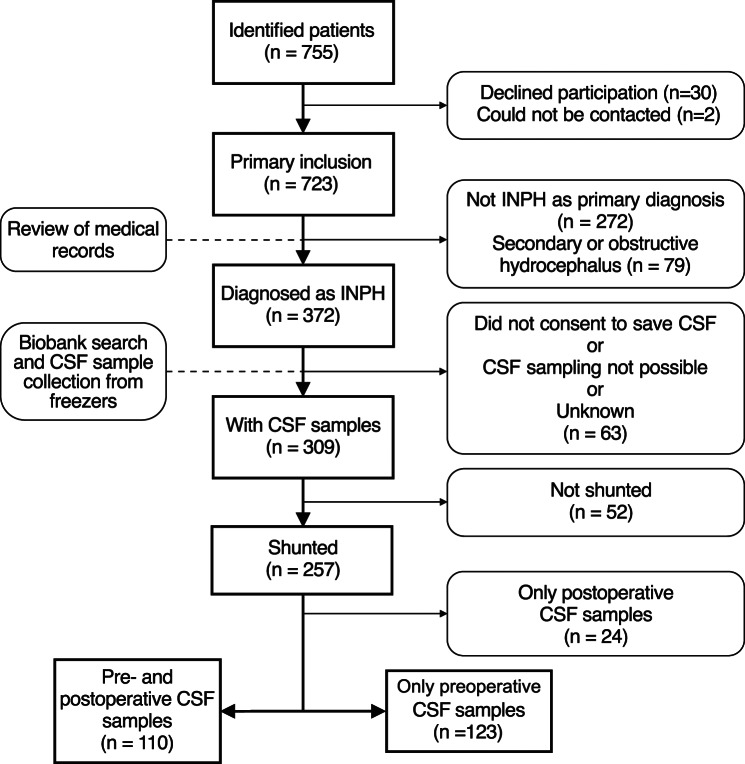
Flowchart of patient inclusion: Patients evaluated for suspected hydrocephalus at our center between 2007 and 2019 were selected for analysis. Abbreviations: Idiopathic normal pressure hydrocephalus (INPH), cerebrospinal fluid (CSF)

The University Hospital of Umeå has a tertiary hydrocephalus unit responsible for investigation, selection for surgery, and CSF shunt surgery. The referral area is northern Sweden, with approximately one million inhabitants. In the present study we included patients with a confirmed diagnosis of probable INPH whose clinical investigation started in 2007–2019 and whose CSF sample was stored in our biobank. Patient flow is shown in Fig. 1.

During the study period, patients referred for ventriculomegaly and associated symptoms underwent a standardized diagnostic work-up that included brain MRI, blood and CSF analyses, CSF infusion testing, a large-volume lumbar tap (the ‘tap test’), and physiotherapist-led assessments of gait and balance. CSF samples were routinely obtained at the time of lumbar puncture, prior to initiation of infusion testing and the tap test. In diagnostically inconclusive cases, a 3-day external lumbar drainage was performed (*n* = 66). Eligibility for shunt surgery required fulfilment of the international criteria for probable INPH [7] and either clinical improvement following CSF drainage and/or pathological CSF dynamic profiles, as determined by consensus at a multidisciplinary conference involving neurologist, neurosurgeon, physiotherapist, nurse, and a biomedical engineer.

Fifty-two individuals diagnosed with INPH were excluded for heterogeneous reasons, including comorbidities making shunt surgery high-risk or unlikely to be beneficial (*n* = 11), declining surgery or further diagnostic evaluation (*n* = 15), marked symptom severity that precluded a reliable assessment of surgical suitability (*n* = 5), negative predictive test results (*n* = 19), and two individuals who died before completing the evaluation.

Shunt function was routinely evaluated as part of standard clinical care 3–6 months after surgery through clinical examination, MRI or CT imaging, and lumbar puncture for infusion testing and CSF analysis to exclude infection. The final study cohort comprised 233 patients with INPH (median age 74 years, IQR 70–78; 37% women). All patients received a ventriculoperitoneal shunt with an adjustable valve, using a standardized opening pressure setting in all but four cases. Twenty-nine patients were not included in the postoperative analyses: Eight underwent shunt removal due to complications or died before follow-up, ten were followed at another center, and eleven declined or were lost to follow-up for unknown reasons. Dropout analysis showed no significant difference in age, sex, centrifugation before storage in freezer, AQP4 levels or any of the available CSF biomarkers.

### Ethical approval

All patients provided written consent to save CSF for future research purposes at the time of lumbar puncture. Participants in this study were informed about the study via a letter, which included an option to opt out if they did not wish to participate. Individuals deceased at the time inclusion began (2020) were automatically incorporated in the cohort. The study was approved by the Swedish ethical review board and the ethical review board at Umeå University. This study was performed in accordance with the guidelines of the Declaration of Helsinki.

### Clinical data

Postoperative gait assessment was available for 204 patients at a median of 5.6 months (IQR 3.7–8.1) after surgery. Among these, 161 patients (79%) underwent infusion testing to verify shunt function; 19 (9%) did not undergo testing, and in 24 (12%) the results were inconclusive. In cases where infusion testing was not performed or yielded inconclusive results, shunt function was evaluated based on clinical assessment and radiological imaging.

CSF samples for biochemical analyses were obtained in 110 and gait was assessed in 107 of these patients at the clinical follow-up, at a median of 4.7 months (IQR 3.5–7.7) after surgery. The postoperative outcome measure was the change in V_max_. Gait velocity was assessed as the mean speed of up to six 10-meter walks observed by a physiotherapist. Patients were classified as shunt responders if V_max_ improved by ≥ 0.16 m/s. This cut-off has been shown to increase the probability of at least one step improvement in the modified Rankin scale [23], i.e., a meaningful improvement for the patient.

Medical records were reviewed to retrieve results for CSF total tau (t-tau), phosphorylated tau (p-tau), amyloid-β42 (Aβ42), and glial fibrillary acidic protein (GFAP), which were analysed at a national laboratory as part of the clinical diagnostic work-up.

During the study period, t-tau, p-tau and Aβ42 were initially analysed using manual quantitative solid-phase enzyme-linked immunosorbent assays (INNOTEST^®^ hTAU Ag, INNOTEST^®^ PHOSPHO-TAU(181P), and INNOTEST^®^ β-AMYLOID(1–42); Fujirebio Europe N.V., Ghent, Belgium). In November 2019, the laboratory transitioned to the automated Lumipulse G 1200 platform (Fujirebio) for these analyses, which also entailed updated reference limits. To ensure methodological consistency, results from three patients analysed after the change of analytical method were excluded from the study.

GFAP was analysed using a manually performed in-house ELISA developed at the laboratory, as previously described [24].

### Handling of the samples and AQP4 determination

Directly after lumbar puncture, 10 ml of CSF was collected in polypropylene tubes. The samples collected before 2012 were stored at -80 °C immediately after collection. The samples collected thereafter were first centrifuged at 400 × g for 10 min before being stored at -80 °C. For controls, 10 ml of CSF was immediately stored at -80 °C after sampling. All CSF samples were stored in the same biobank.

AQP4 CSF measurements were performed at SciLifeLab in Stockholm via an antibody- and bead-based technique [25] with a polyclonal rabbit IgG antibody (HPA014784) produced within the Human Protein Atlas project (HPA, www.proteinatlas.org). In brief, the antibody was immobilized onto the surface of magnetic carboxylated beads. Fifteen microliters of each sample were diluted 1/2 into 96-well plates before labelling with a tenfold molar excess of biotin (21329, Thermo Scientific) as described previously [25]. Detection of captured AQP4 was enabled by the addition of a streptavidin-coupled fluorophore (SA1004-4; Invitrogen). Read-out was performed in a FlexMap3D instrument (Luminex Corporation) and the results reported as median fluorescence intensities per bead identity and sample, calculated from at least 30 measured beads. To evaluate the technical variability of the assay, coefficients of variation were calculated across all replicate samples and determined to be 4%. More detailed explanations of the experimental procedures can be found elsewhere [25]. Antibody specificity was evaluated in previous studies [26, 27].

### Statistical analysis

Statistical analyses were conducted using IBM SPSS version 28.01.1 (SPSS, IBM Software Group, Chicago, IL, USA). Continuous variables are presented as mean ± SD or median (interquartile range, IQR), as appropriate, determined by tests of normality (Kolmogorov–Smirnov and Shapiro–Wilk). Group comparisons were performed using parametric or non-parametric tests depending on data distribution. Group differences in preoperative AQP4 levels between INPH patients and controls, as well as between shunt responders and non-responders, were examined using a generalized linear model (GLM) adjusting for age, sex, sample centrifugation prior to storage, and storage duration in the freezer. Correlations were assessed with Pearson’s (r) or Spearman’s (r_s_) coefficients depending on variable type and distribution. A p-value < 0.05 was considered statistically significant.

## Results

### Sample and patient characteristics

Table 1 summarizes the sample characteristics of patients with INPH and controls. The groups were matched for age, although a male predominance was observed in the INPH group. Notable differences were identified in terms of sample processing and storage conditions. Specifically, 75% of the INPH samples underwent centrifugation prior to storage, whereas none of the control samples were centrifuged. Furthermore, there was a discrepancy in the storage duration, with INPH samples collected between 2007 and 2022, compared with the control samples, which were collected between 2003 and 2004.

**Table 1:** Characteristics of samples from INPH patients and controls

	INPH	Control	*P* value
Participants (*n*)	233	29	*N*/A
Age (years) median (IQR)	74 (70–78)	72 (69–78)	0.36^†^
Female (%)	37	62	0.0078^‡^
Centrifuged prior to storage (%)	75	0	N/A
Storage time (days) median (IQR)	2689 (1667–4016)	6702 (6647–6828)	< 0.001^†^

† = Independent samples Mann-Whitney U test. ‡ = Pearson Chi-Square (two-sided). Abbreviations: Idiopathic normal pressure hydrocephalus (INPH), interquartile range (IQR)

### CSF AQP4 levels in INPH patients versus controls

**Fig. 2:**
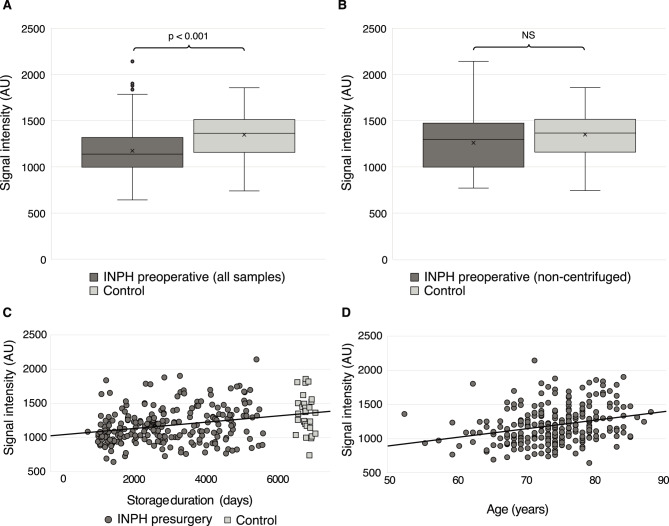
**A.** CSF AQP4 levels among all preoperative INPH samples (*n* = 233) compared with non-centrifuged controls (*n* = 29). **B.** CSF AQP4 levels among non-centrifuged preoperative INPH samples (*n* = 59) compared with non-centrifuged controls (*n* = 29). **C**. Correlation between CSF AQP4 levels and freezer storage duration across all preoperative INPH samples and controls (*n* = 262). A positive correlation was observed (*r* = 0.30, *p* < 0.001). **D.** Correlation between CSF AQP4 levels and age across all preoperative INPH samples and controls (*n* = 262). A positive correlation was observed (*r* = 0.28, *p* < 0.001). The values are in arbitrary units (AUs). Abbreviations: Aquaporin-4 (AQP4); idiopathic normal pressure hydrocephalus (INPH); not significant (NS)

In summary we could not find any difference between INPH patients and controls. Without adjustment for sex or pre-analytical sample handling, preoperative INPH patients showed lower AQP4 levels (median 1142 AU, IQR 1000–1321) compared with controls (median 1366 AU, IQR 1160–1516; *p* < 0.001) (Fig. 2A). However, to examine group differences while accounting for covariates, we conducted a generalized linear model with AQP4 levels as the dependent variable and diagnostic group (INPH vs. control) as the predictor. The model adjusted for sex (*p* = 0.13), centrifugation prior to storage (*p* = 0.87), storage duration (*p* = 0.004), and age (*p* < 0.001). After adjustment, no difference in AQP4 levels was found between INPH patients and controls (*p* = 0.87). This is in accordance with analysis of unadjusted data where, positive correlations were observed between AQP4 levels and storage duration (*r* = 0.30, *p* < 0.001) (Fig. 2C), as well as between AQP4 levels and age across INPH patients and controls (*r* = 0.28, *p* < 0.001) (Fig. 2D).

In addition, we repeated the analysis including only the homogenous group of non-centrifuged preoperative samples with storage times more comparable to controls (*n* = 59). In this restricted comparison, no significant difference was observed between INPH patients (mean 1261 ± 303 AU) and controls (mean 1351 ± 279 AU; *p* = 0.17) (Fig. 2B). This non-significant finding remained after controlling for age, sex and storage time in freezer.

### Postoperative CSF AQP4 levels

**Fig. 3:**
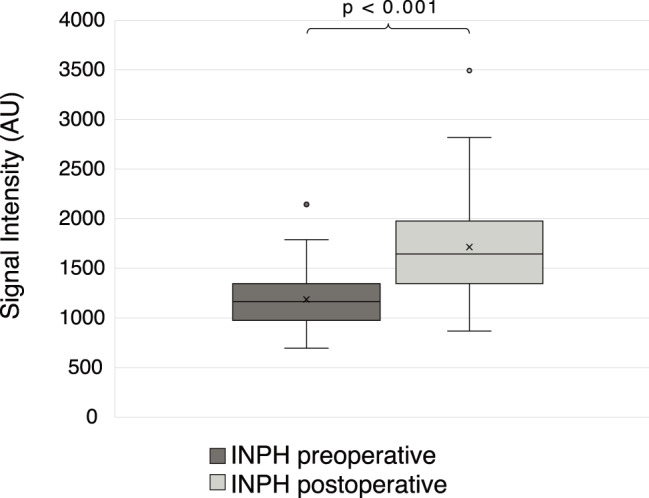
Preoperative and postoperative CSF AQP4 levels in INPH patients (*n* = 110). The values are presented in arbitrary units (AUs). Postoperative CSF samples were obtained by lumbar puncture at a median of 4.7 (IQR 3.5–7.7) months after surgery. Abbreviations: Aquaporin-4 (AQP4); idiopathic normal pressure hydrocephalus (INPH)

In the longitudinal follow-up of INPH patients (*n* = 110), we observed an increase in AQP4 levels after surgery (median 1646 AU IQR 1347–1976) compared with the preoperative levels (median 1166 AU IQR: 976–1345; *p* < 0.001) (Fig. 3). Only nine out of the 110 patients presented reduced AQP4 levels at follow-up. In a subgroup analysis including only samples with identical pre- and postoperative preanalytical handling, the difference remained significant (*p* < 0.001, *n* = 102).

### CSF AQP4 levels in shunt responders

**Fig. 4:**
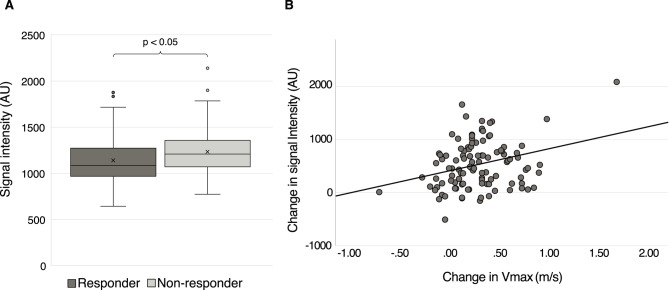
**(A)** Preoperative CSF AQP4 levels in shunt responders (*n* = 130) compared with non-responders (*n* = 74). Postoperative gait was assessed at a median of 5.6 months (IQR 3.7–8.1) after surgery. **(B)** Correlation between the preoperative-to-postoperative change in CSF AQP4 levels and the preoperative-to-postoperative change in V_max_ in INPH patients (*n* = 107). A weak positive correlation was observed (rₛ = 0.22, *p* = 0.022). Postoperative CSF samples were obtained by lumbar puncture, and gait was assessed at a median of 4.7 (IQR 3.5–7.7) months after surgery. The values are presented in arbitrary units (AUs). Abbreviations: Idiopathic normal pressure hydrocephalus (INPH); maximal gait velocity (V_max_)

Among the 233 INPH patients with preoperative samples, postoperative V_max_ data were available in 204. Of these, 130 patients (64%) were classified as shunt responders. Preoperative AQP4 levels were lower in responders (median 1089 AU, IQR 971–1277) than in non-responders (median 1213 AU, IQR 1074–1361; *p* = 0.008) (Fig. 4A). When restricting the analysis to centrifuged samples, the group difference remained significant (*p* = 0.023, *n* = 149). In the full cohort, the difference likewise remained significant after adjusting for age, sex, storage duration, and pre-freezing centrifugation in a GLM analysis (*p* = 0.023, *n* = 204). In a corresponding GLM restricted to centrifuged samples, the group difference approached significance (*p* = 0.07, *n* = 149).

For the full cohort (Table 2), responders and non-responders did not differ in age, sex, sample centrifugation prior to storage, storage duration, Mini-Mental State Examination score, implanted shunt type, or shunt opening pressure. In the subgroups with available CSF biomarkers (t-tau, p-tau. Aβ42 and GFAP), non-responders had significantly higher t-tau and p-tau concentrations, although the proportion of patients above the predefined clinical cut-offs did not differ between groups.

In the 107 patients with both pre- and postoperative samples and postoperative V_max_ data, the change in AQP4 levels from pre- to postoperative measurements was weakly positively correlated with the change in postoperative V_max_ (r_s_ = 0.22, *p* = 0.022) (Fig. 4B). This association remained in a subgroup analysis restricted to samples with same pre- and postanalytical handling (r_s_ = 0.24, *p* = 0.017, *n* = 99).

### Dropout analyses

Patients with negative predictive test results (*n* = 19) and patients excluded for other reasons (*n* = 33) were each compared with shunt responders (*n* = 130). No differences were observed in median preoperative CSF AQP4 levels, age, sex distribution, storage duration, or centrifugation status between groups. A non-significant trend toward higher CSF AQP4 levels in patients with negative predictive test results compared with shunt responders was observed (*p* = 0.077).

**Table 2:** Patient and sample characteristics of shunt responders and non-responders

	Shunt responder		*P*-value
	Yes	No	
**Number** ^**†**^	130	74	N/A
**Age (years) median (IQR)**	74 (70–78)	76 (70–78)	0.63^‡^
**Female (%)**	37	32	0.55^§^
**Centrifuged before storage (%)**	72	76	0.62^§^
**Storage duration (days) median (IQR)**	2539 (1477–4115)	2741 (2072–3919)	0.38^‡^
**CSF AQP4 levels (AU) median (IQR)**	1089 (971–1277)	1213 (1074–1361)	**0.008** ^‡^
**Change in V**_**max**_ **(m/s) median (IQR)**	0.37 (0.23–0.61)	-0,02 (-0.16–0.08)	**< 0.001** ^‡^
**MMSE score median (IQR)** (***n*** **= 197)**	26 (21–28)	27 (24–28)	0.15^‡^
**Shunt type (%)**:			
Medtronic Strata™	91.5	94.6	0.86^¶^
Medtronic Strata MR™	7.7	5.4	
Codman Certas^®^ Plus	0.8	0.0	
**Strata shunt opening pressure (%)**^**¤**^:			
1.5	98.4	98.6	1.0^¶^
> 1.5	1.6	1.4	
**CSF concentration median (ng/L) (IQR)**:			
Tau (n_yes_ = 107; n_no_ = 60)	198 (158–267)	230 (201–310)	**0.014** ^**‡**^
p-Tau (n_yes_ = 103; n_no_ = 57)	30 (24–38)	37 (29–49)	**0.003** ^**‡**^
Aβ42 (n_yes_ = 108; n_no_ = 60)	421 (330–534)	451 (337–576)	0.42^‡^
GFAP (n_yes_ = 98; n_no_ = 59)	490 (350–690)	570 (400–970)	0.15^‡^
**Tau > cut-off (400 ng/L) (%)**	6.4	8.1	0.76^¶^
**p-Tau > cut-off (80 ng/L) (%)**	0.9	5.1	0.13^¶^

Shunt responder = ≥ 0.16 m/s improvement in maximal gait velocity after shunt surgery. † = Data for improvement in maximal gait velocity was missing from 29 out of 233 patients with preoperative samples. ‡ = Independent-Samples Mann-Whitney U test. § = Pearson Chi-Square test, (two-sided). ¶ = Fisher’s exact test, (two-sided). ¤ = Data for shunt opening pressure was missing from one out of 130 shunt responders. Abbreviations: IQR = interquartile range; AU = Arbitrary Units; V_max_ = maximal gait velocity; p-Tau = phosphorylated tau; Aβ42 = beta-amyloid 42; GFAP = glial fibrillary acidic protein; n_yes_ = shunt responder; n_no_ = non-responder

## Discussion

CSF AQP4 levels were quantified in a large INPH cohort. After adjusting for pre-analytical factors and demographic differences, AQP4 levels in INPH patients were comparable to those of neurologically healthy controls. Furthermore, AQP4 levels increased postoperatively, and higher postoperative levels showed a weak positive association with clinical improvement. Preoperative AQP4 levels were lower in shunt responders than in non-responders.

### Pre-analytical factors and interpretation of CSF AQP4

Our findings demonstrate that CSF AQP4 levels can be reliably quantified using a suspension bead array in a large INPH cohort. Storage duration and sample processing exerted a strong influence on measured CSF AQP4 levels, underscoring the importance of accounting for these variables when analysing and interpreting results. These findings highlight the need for harmonized preanalytical procedures in future studies to enable more precise evaluation of AQP4 as a potential biomarker in INPH.

### CSF AQP4 and glymphatic function in INPH

If CSF AQP4 reflects overall astrocytic expression, and our findings are not related to confounding factors, this would suggest that global AQP4 expression is not markedly reduced in INPH. However, altered perivascular localization of AQP4—a key determinant of glymphatic flow [2, 3, 14]—could still be present without affecting total CSF levels. Therefore, CSF AQP4 alone might not capture the more nuanced astrocytic alterations implicated in glymphatic dysfunction. However, CSF AQP4 levels within the normal range do not support glymphatic dysfunction. These findings contrast with previous MRI studies reporting delayed gadolinium-based contrast enhancement in INPH, interpreted as evidence of reduced glymphatic clearance [11, 12]. Yet, such delays may also arise from known disturbances in CSF circulation in INPH, including elevated outflow resistance [8] and disproportionate enlargement of the ventricles and SAS [28]. Studies combining CSF AQP4 measurements with MRI-based tracer dynamics in the same cohort would be required to clarify the relationship between these approaches.

### AQP4 – from the plasma membrane to CSF

The cellular mechanisms regulating AQP4 expression and its turnover into CSF remain poorly understood [1]. One recent study revealed that membrane-bound AQP4 is continuously recycled or internalized through vesicular trafficking [29]. Membrane-bound proteins are typically internalized and degraded via the ubiquitin‒lysosome pathway [30], suggesting that this mechanism likely governs AQP4 degradation in astrocytes. Consequently, AQP4 is not expected to leave astrocytes under normal circumstances. However, the detection of AQP4 in CSF raises the possibility that its turnover between astrocytes and CSF is mediated by additional processes, potentially involving the release of microvesicles or exosomes [31]. An alternative explanation is that AQP4 enters the ISF or CSF because of astrocytic cell injury. However, histopathological studies in INPH have not demonstrated overt astrocytic injury or necrosis. Instead, reactive astrogliosis has consistently been reported as a prominent finding [14–16], suggesting that the presence of AQP4 in CSF is less likely to result from passive leakage due to cellular damage and may instead reflect regulated or secondary processes. In summary, the exact form in which AQP4 is measured in CSF remains unclear and warrants further investigation.

### Previous studies of CSF AQP4 levels in INPH, AD and FTD

As previously mentioned, two studies have investigated AQP4 in CSF from INPH patients. The first found no difference in AQP4 levels between INPH and controls; however, this study included only 10 subjects with INPH and 9 controls. A second, larger study failed to detect any AQP4 in CSF, raising concerns about potential methodological issues. Taken together with our results, studies so far suggest that AQP4 levels in CSF are similar in INPH patients and controls.

AQP4 has been more extensively investigated in AD, which has been proposed to share several features with INPH in terms of glymphatic dysfunction [10, 16]. Arighi et al. initially reported lower AQP4 levels in the CSF in a small study including eleven subjects with AD and nine controls [21]; however, in a subsequent larger study, they reported contradictory results, with increased levels [32]. Bergström et al. [26] conducted a multicentre study comparing AQP4 levels in AD patients and controls and reported elevated AQP4 levels in CSF of AD patients across all four studied cohorts. These findings were recently corroborated by another study in which CSF AQP4 levels were quantified via ELISA [27]. Notably, increased AQP4 levels in CSF have also been reported in frontotemporal dementia (FTD) patients [33].

If CSF AQP4 levels are elevated in AD and FTD but remain within the normal range in INPH, this may suggest that the underlying mechanisms contributing to fluid-flow disturbances differ between these disorders. AQP4 may therefore have value as a biomarker for detecting astrocytic dysregulation in conditions such as AD and FTD, whereas such alterations were not evident in INPH in the present cohort.

### CSF AQP4 levels and shunt surgery

We observed a general increase in CSF AQP4 levels following shunt surgery, with responders displaying lower preoperative levels than non-responders. Moreover, postoperative increases in AQP4 levels correlated weakly with clinical improvement, as measured by changes in V_max_.

One possible explanation for the postoperative rise in CSF AQP4 is a concentration effect due to reduced CSF volume. However, earlier studies of other potential CSF biomarkers in INPH, including amyloid-β42, t-tau, and p-tau [34] and ten different proteins [35] did not identify significant concentration effects from reduced CSF ventricular volume after shunt surgery. Two Japanese groups have investigated changes in intracranial brain and CSF volumes after shunt surgery, observing primarily a redistribution of CSF between different compartments of the SAS, with only minor reductions in total intracranial CSF volume at 1–12 months postoperatively (11% and 5%, respectively) [36, 37]. These modest changes are unlikely to explain the increase in AQP4 levels observed here.

Another potential mechanism is a sedimentation effect along the cranial–lumbar axis due to altered CSF circulation caused by shunting from the ventricles near the choroid plexus. Yet shunt surgery has been shown to produce variable effects across CSF proteins, with both increases and decreases in lumbar CSF concentrations [35] making predictions for AQP4 behaviour uncertain, particularly in the absence of ventricular CSF samples.

A further hypothesis is that elevated postoperative AQP4 levels reflect altered glymphatic flow due to decreased intracranial pressure and increased compliance after shunt surgery. Such changes might stimulate AQP4 expression in astrocytes and/or enhance recirculation of ISF, promoting transport of proteins, including non–membrane-bound AQP4, into CSF.

The finding that responders had lower preoperative CSF AQP4 levels than non-responders, together with the weak correlation between improvement in V_max_ and postoperative AQP4 changes, is intriguing. Shunt responders and non-responders differed in T-tau and p-tau levels, however the proportion of patients exceeding clinically established threshold values did not differ between the groups. This limits the clinical interpretability of the group differences. Nonetheless, and acknowledging the speculative nature of this interpretation, the higher tau and p-tau concentrations observed in non-responders may still reflect a greater burden of comorbid neurodegeneration, consistent with previous reports of elevated CSF AQP4 levels in AD and FTD [26, 27, 32, 33]. INPH is a heterogeneous condition often accompanied by neurodegenerative comorbidities such as subcortical vascular dementia, parkinsonism, and AD. Responders in our study may therefore represent a comparatively ‘purer’ INPH phenotype, with less neurodegenerative pathology and consequently comparatively lower or normal preoperative AQP4 levels. Supporting this interpretation, dropout analysis revealed a trend toward higher CSF AQP4 levels among the patients who were excluded from shunt surgery on the basis of negative tap tests and normal outflow resistance. Furthermore, in INPH patients, the disturbed CSF dynamics typical of the disease were improved by shunting, leading to clinical improvement. As a sign of a functioning shunt, this change in CSF circulation also resulted in a postoperative rise in AQP4, likely due to mechanisms previously discussed.

In summary, this could suggest that INPH entails a CSF disturbance that may not be a AQP4-related glymphatic dysfunction. At the same time, it supports the notion that elevated AQP4 levels may indicate comorbid neurodegenerative disease, which is known to be associated with higher levels of AQP4 in CSF [26, 27, 32, 33] and reduced clinical effect from CSF drainage [38].

### Strengths and weaknesses

This study provides important data on CSF AQP4 levels in a large INPH cohort and benefits from detailed postoperative follow-up, including a substantial number of patients who underwent postoperative lumbar punctures and infusion testing to verify shunt function. Several limitations should, however, be considered. Pre-analytical handling varied, as control samples were collected several years earlier than INPH samples, and a proportion of INPH samples underwent centrifugation prior to storage. Storage duration among INPH samples (2007–2019) also varied considerably, introducing heterogeneity that affected AQP4 quantification. In addition, the control group was relatively small and contained a higher proportion of females, which may have influenced group comparisons. Therefore, although differences in pre-analytical methods, age, and sex were accounted for in the statistical analyses, comparisons between INPH patients and controls should still be interpreted with caution.

## Conclusion

In summary, we found no evidence of altered CSF AQP4 levels in INPH patients once pre-analytical variation was accounted for. The strong influence of pre-analytical variation indicates that standardized CSF handling will be essential for future studies of AQP4. The postoperative rise in AQP4 and its modest association with clinical improvement, together with lower preoperative levels in shunt responders, indicate that AQP4 may hold relevance for understanding treatment-related physiological changes and for identifying shunt-responsive patients. Findings from this study motivate future studies, employing standardized and tightly controlled CSF handling protocols to clarify the biological role of AQP4 in CSF and its potential diagnostic or prognostic utility in INPH.

## Data Availability

The datasets used and/or analyzed during the current study are available from the corresponding author on reasonable request.

## Abbreviations

AQP4: Aquaporin 4
CSF: Cerebrospinal fluid
INPH: Idiopathic normal pressure hydrocephalus
SAS: Subarachnoid space
ISF: Interstitial fluid
MRI: Magnetic resonance imaging
AD: Alzheimer’s disease
V_max_: Maximal gait velocity
t-tau: Total tau
p-tau: Phosphorylated tau
Aβ42: Amyloid-β42
GFAP: Glial fibrillary acidic protein
IQR: Interquartile range
HPA: Human Protein Atlas
AU: Arbitrary Units
MMSE: Mini-Mental State Examination
FTD: Frontotemporal dementia
